# A review on advances in ^18^F-FDG PET/CT radiomics standardisation and application in lung disease management

**DOI:** 10.1186/s13244-021-01153-9

**Published:** 2022-02-05

**Authors:** Noushin Anan, Rafidah Zainon, Mahbubunnabi Tamal

**Affiliations:** 1 Department of Biomedical Imaging, Advanced Medical and Dental Institute, Universiti Sains Malaysia, SAINS@BERTAM, 13200 Kepala Batas, Pulau Pinang Malaysia; 2 School of Physics, Universiti Sains Malaysia, 11800 Gelugor, Pulau Pinang Malaysia; 3 Department of Biomedical Engineering, College of Engineering, Imam Abdulrahman Bin Faisal University, PO Box 1982, Dammam, 31441 Saudi Arabia

**Keywords:** ^18^F-FDG PET/CT, Biomarker, Lung diseases, Radiomics feature, Standardisation

## Abstract

Radiomics analysis quantifies the interpolation of multiple and invisible molecular features present in diagnostic and therapeutic images. Implementation of 18-fluorine-fluorodeoxyglucose positron emission tomography/computed tomography (^18^F-FDG PET/CT) radiomics captures various disorders in non-invasive and high-throughput manner. ^18^F-FDG PET/CT accurately identifies the metabolic and anatomical changes during cancer progression. Therefore, the application of ^18^F-FDG PET/CT in the field of oncology is well established. Clinical application of ^18^F-FDG PET/CT radiomics in lung infection and inflammation is also an emerging field. Combination of bioinformatics approaches or textual analysis allows radiomics to extract additional information to predict cell biology at the micro-level. However, radiomics texture analysis is affected by several factors associated with image acquisition and processing. At present, researchers are working on mitigating these interrupters and developing standardised workflow for texture biomarker establishment. This review article focuses on the application of ^18^F-FDG PET/CT in detecting lung diseases specifically on cancer, infection and inflammation. An overview of different approaches and challenges encountered on standardisation of ^18^F-FDG PET/CT technique has also been highlighted. The review article provides insights about radiomics standardisation and application of ^18^F-FDG PET/CT in lung disease management.

## Key points

- The micro-level changes in lung disease cannot be detected by CT alone.
- Employment of ^18^F-FDG PET/CT radiomics moves toward patient-specific of lung diseases management.
- However, numerous features and techniques of feature extraction have raised tremendous complexity.
- This complexity can only be removed by standardisation of radiomics analysis.
- The artificial intelligence and machine learning with radiomics analysis improve diagnosis and treatment.

## Background

Molecular haracterisation of physiological abnormality is conventionally performed by biopsy or random sample collection of the suspected site for diagnosis and identification. However, biopsy is an invasive process, and it has risk of complication such as infection [1, 2]. Lung cancer is one of the leading causes of respiratory morbidity and mortality among adults [3]. Thus, to avoid this risk, medical imaging techniques, for instance magnetic resonance imaging (MRI), computed tomography (CT) and positron emission tomography (PET), and hybrid imaging modalities such as PET/CT and PET/MRI are widely used to determine the tumour heterogeneity and morphological abnormalities in a non-invasive manner [4, 5]. ^18^F-Fluorodeoxyglucose (^18^F-FDG) PET/CT is one of the prevailing diagnostic tools, especially in the field of oncology and other clinical disorders owing to its higher accuracy in diagnosis, prognosis and therapeutic response assessment [6–11]. Lung cancer is one of the primary causes of death around the world [12]. The application of ^18^F-FDG PET/CT is recognised to reduce the morbidity rate [13]. PET imaging has also gathered attention in the field of neuroimaging and cardiac imaging and vascular abnormality detection [14–16].

Medical images have the ability to capture cellular- and molecular-level tumour characteristics reflected in phenotype [17–20]. A study on CT image of anaplastic lymphoma kinase mutations in lung tumours captured substantial pulmonary fluid and no pulmonary tails [21]. In another study, CT images of complementary contrast demonstrated that the metamorphosis of von Hippel–Lindau located in renal cell carcinoma is considerably correlated with the total intra-tumoral vasculariSation, sharp edges of the tumour and nodular advancement of the tumour [22]. Conventionally, CT scan is performed on different stages of cancer treatment to understand the drug efficacy [23]. Before surgery, the severity of lung cancer is assessed performing therapeutic techniques and invasive diagnosis. These are termed as conventional workup. Integration of ^18^F-FDG PET with therapeutic techniques and invasive diagnosis leads to 51% consequential decline of impractical thoracotomy such as eliminating one in five critical surgeries comprehensively when compared with conventional workup exclusively, in the PET in lung cancer staging multicentre randomised trial [24].

Rise of glucose metabolism is a well-recognised hallmark of cancer, and molecular PET imaging mainly captures these increased metabolism for diagnostic purpose [25]. Cell sites exposed to abnormal proliferation, infection and inflammation can be determined by identifying the high uptake and accumulation of the glucose analogue, ^18^F-FDG [13, 26]. ^18^F-FDG combining with the glucose transporters present in cell and phosphorylated by hexokinase results in ^18^F-FDG-6-phosphate misses 2-hydroxyl group needed for glycolysis [13, 27, 28]. This complex chemical component remains metabolically trapped in the cell and can be detected through PET imaging [13, 14, 29]. ^18^F-FDG uptake in PET imaging depends on the number of active cancer cells, histopathology of tumour, and biological processes responsible for continuous oncogenesis [30–33]. Therefore, studies have shown that heterogeneity of tumour may be correlated with the sparse distribution ^18^F-FDG distribution [17, 34, 35]. The ^18^F-FDG PET has been proven to have greater mediastinal staging capability than CT in according to a meta-analysis study [36]. Furthermore, ^18^F-FDG PET/CT produces more precise image quantification information compared to either imaging modality individually [37].

Radiomics analysis is an emerging field in the medical imaging sector, and it is recognised as a promising classification tool that holds the innate potential of revolutionising disease diagnosis specially cancer [38–42]. Radiomics has been introduced in imaging field to strengthen the conventional and manual image comprehension by recognising features and patterns, which largely remains undetected to the human eye [43, 44]. Radiomics enables extraction, collection and evaluation of higher order and statistical datasets through radiographic information conversion into large-scale and mineable entities [44–47]. Generally, radiomics analysis process is impartial to the disease under investigation, and it is performed in the order of data acquisition, data pre-filtration, region of interest (ROI) selection, feature derivation, post-filtration following data investigation [37]. The field of radiomics mainly targets improving patient management such as disease-type prediction, survival rate and efficacy of therapy [45, 48–55]. Detailed investigation of single nodule alongside more nodules within the region of interest in nearly real-time result production is some of the many technical advantages of radiomics [56]. Previous studies focused on the development and validation of machine-learning-based clinical models to predict the patient outcome to ensure that it becomes feasible and practical [54]. However, these multicentric models consist of enormous amount of higher-order and diverse functionality-based image features; as a result, interpreting and understanding these features become overwhelming even for the experts in the field [57, 58]. At present, researchers are working on upgrading the readability of quantitative information of the radiomics model so that radiologists and physicians can comprehend the data effortlessly [59]. To replace the current diagnostic assessment of imaging technique (human eye interpretation), the features must be accurate, robust and reproducible. Dedicated research works are performed to attain this goal, and some of the published works have very inspiring results [53, 59, 60].

Radiomics can serve as biomarkers, incorporated with artificial intelligence (AI), and it can be used to develop prediction models that may enable a far more detailed, precise and micro-level assessment, well beyond the predominantly methodological techniques used in medical image evaluation at present [42, 61]. The ^18^F-FDG PET as a potential biomarker for therapeutic response evaluation was acknowledged way back in 1999 [62]. The ^18^F-FDG PET/CT biomarkers facilitate immunotherapy response prediction in advanced stages of non-small cell lung cancer (NSCLC) [63]. However, ubiquitous establishment and recognition of ^18^F-FDG PET/CT as a computable biomarker are lacking due to the absence of standardised imaging and data exploration techniques [64]. On a brighter side, literature review confirms that researches are working on overcoming these challenges; thus, the ^18^F-FDG PET/CT can be established as a quantitative biomarker in near future [56, 61].

**Fig. 1:**
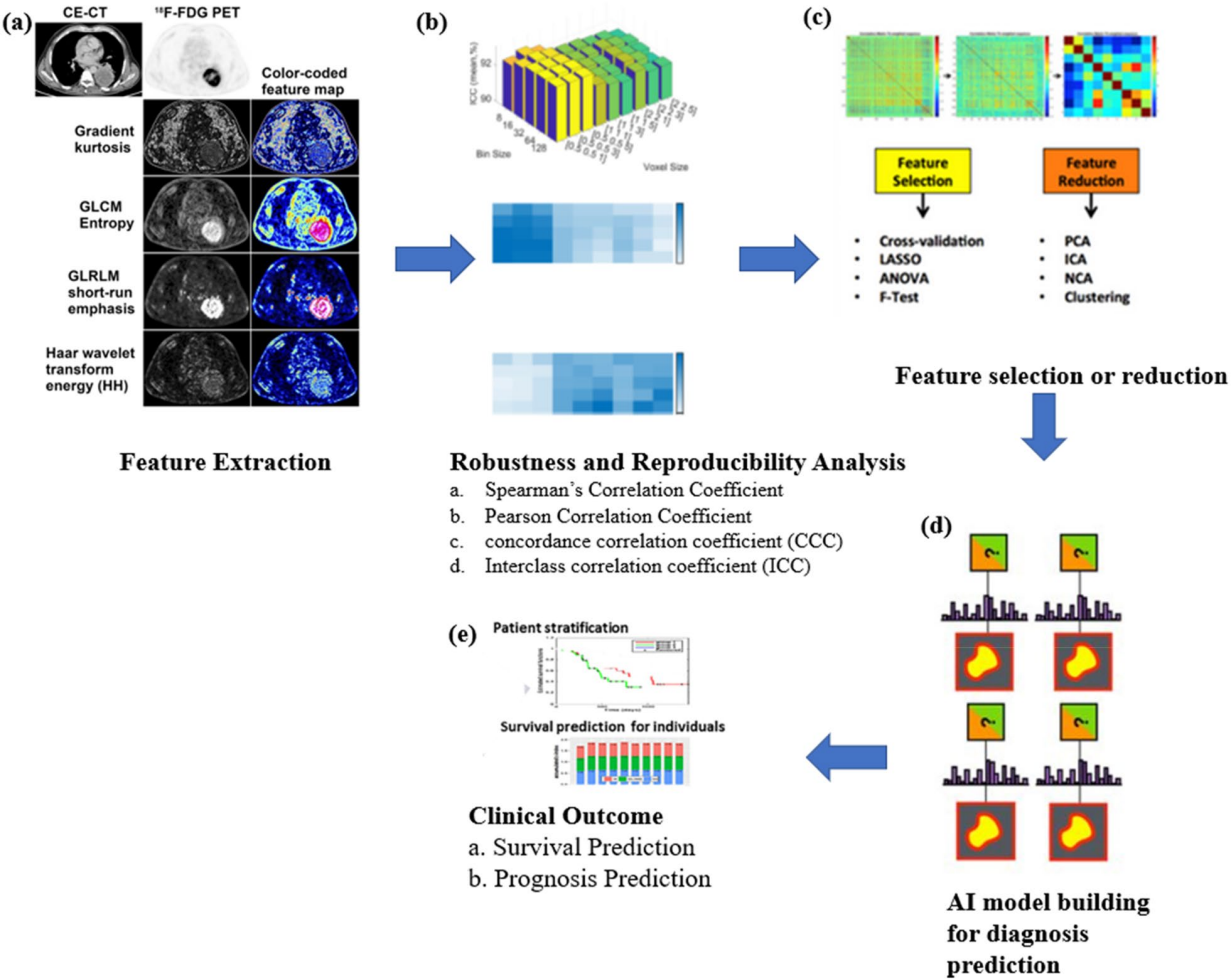
The overview of optimisation of radiomics feature for its translation in clinical practice

Transition of radiomics finding into therapeutic practice is the ultimate goal of the field of radiomics and texture analysis. Figure 1 shows the overview of optimisation of radiomics feature for clinical practice translation. The process begins with feature extraction from the region of interest through a computerised method (Fig. 1a) [65]. Next, robustness and the reproducibility of the features are determined by evaluating Spearman’s correlation coefficient, Pearson correlation coefficient, concordance correlation coefficient or interclass correlation coefficient (Fig. 1b). Based on the robustness and reproducibility findings, the optimum features are selected and redundant features are removed (Fig. 1c) [66]. For automisation of the clinical practice, artificial intelligence-based model is developed for lung disease prediction, prognosis and diagnosis (Fig. 1d) [65]. Finally, clinical outcomes such as survival prediction and prognosis prediction can be achieved by the application of radiomics (Fig. 1e) [67].

In this review paper, we discuss the significant aspects of ^18^F-FDG PET/CT radiomics in proper management of lung diseases (cancer, infection and inflammation) and standardisation initiatives, progress and challenges. This review article is written appreciating the importance of ^18^F-FDG PET/CT so that it can be recognised as quantitative biomarker. The article has been divided into two parts: The first part addresses the application of radiomics in assessing and diagnosing lung diseases and the factors affection radiomics analysis. The second part of this paper addresses the challenges face in standardisation of radiomics features and approaches involving standardisation of morphological, textural and statistical radiomics features.

## Application of ^18^F-FDG PET/CT radiomics

### Lung cancer detection and assessment

Cancer is the uncontrolled cell proliferation that follows death without proper detection, assessment and treatment during the earlier stages [68]. Application of PET in the field of oncology has been well recognised as PET imaging has the ability to extract phenotypic and functional tumour heterogeneity information [61]. During cancer treatment, the metabolic transformation usually takes place before anatomical changes, and this can be spotted through ^18^F-FDG PET [69]. Commonly used ^18^F-FDG PET/CT parameters such as standardised uptake value derivatives, metabolically active tumour volume and total lesion glycolysis have widespread application in oncological medicine [70, 71]. Tumours tend to have intricate topological arrangement termed as intra-tumour heterogeneity, and tumour phenotype assessment by intra-tumour heterogeneity quantification is beyond the scope of these matrices [71]. Prediction models have been established to distinguish benign, malignant and inflammatory pulmonary condition emphasising computable imaging features [42, 72–75]. Texture analysis of radiotracer uptake value significantly increased specificity compared to maximum standardised uptake value alone [7, 76]. Texture analysis has been adopted for primary and metastatic lesions distinction on a considerably large patient cohort (*n* = 545) in a retrospective study [77].

In the field of oncology, researchers have acknowledged the exceptional tumour-to-background ratio and greater tumour examination capacity of ^18^F-FDG PET/CT compared to CT imaging [16]. Investigation on medical application of ^18^F-FDG PET/CT in oncological patients showed that high concentration of ^18^F-FDG in pulmonary nodules might represent malignancy of the investigated lesion [78]. Among lung cancer variations, NSCLC holds major concern [79]. Adenocarcinoma, squamous cell carcinoma and large cell carcinoma are its primary subtypes [37]. Healthcare professionals, radiologists and scientists are working hand in hand to overcome its danger and trying to manage cancer risk by extracting and interpreting information from these associating histological subtypes, micro-level tumour characteristics and understanding the tumour stage using CT and PET radiomic signatures [37, 71, 80, 81]. ^18^F-FDG PET facilitates detailed apprehension and evaluation of carcinoma traits such as its metabolism and receptor recognition on the microscopic level and so its application in NSCLC patient management is clinically momentous [37]. ^18^F-FDG PET/CT has received appreciation from the National Comprehensive Cancer Network for having NSCLC patient evaluation capability [82]. American College of Radiology Appropriateness Criteria and American College of Chest Physicians guidelines have also recommended ^18^F-FDG-PET/CT for NSCLC staging due to its well-recognised effectiveness [83, 84]. Minuscule ^18^F-FDG uptake by subcentimetre pulmonary nodules was reported to be benign in 98% cases in a study performed on large cohort of population [71, 85]. Abatement of tumour size detection might be extremely slow or remain hidden in the targeted drugs; for example, thymidine kinase inhibitors’ treatment period and evaluation of the competence these drugs can be done earlier by ^18^F-FDG PET/CT [86]. Computable ^18^F-FDG PET/CT investigation studies might promote observer-independent appraisal of tracer uptake, thus expanding its capacity of turning into image biomarker [87, 88]. Moreover, combination of CT and ^18^F-FDG PET enhances the NSCLC patient management by combining anatomic and biologic information [89].

Texture analysis is a specialised branch of radiomics concentrating on quantitative analysis and regional topology variation discretisation of the image voxel densities [47, 90, 91]. Initially, researchers became enthusiastic about texture when they realised that phenotypic characteristics present in diagnostic images can be distinguished though higher-order statistical aspects remain unidentified by visual perception alone [91, 92].

**Fig. 2:**
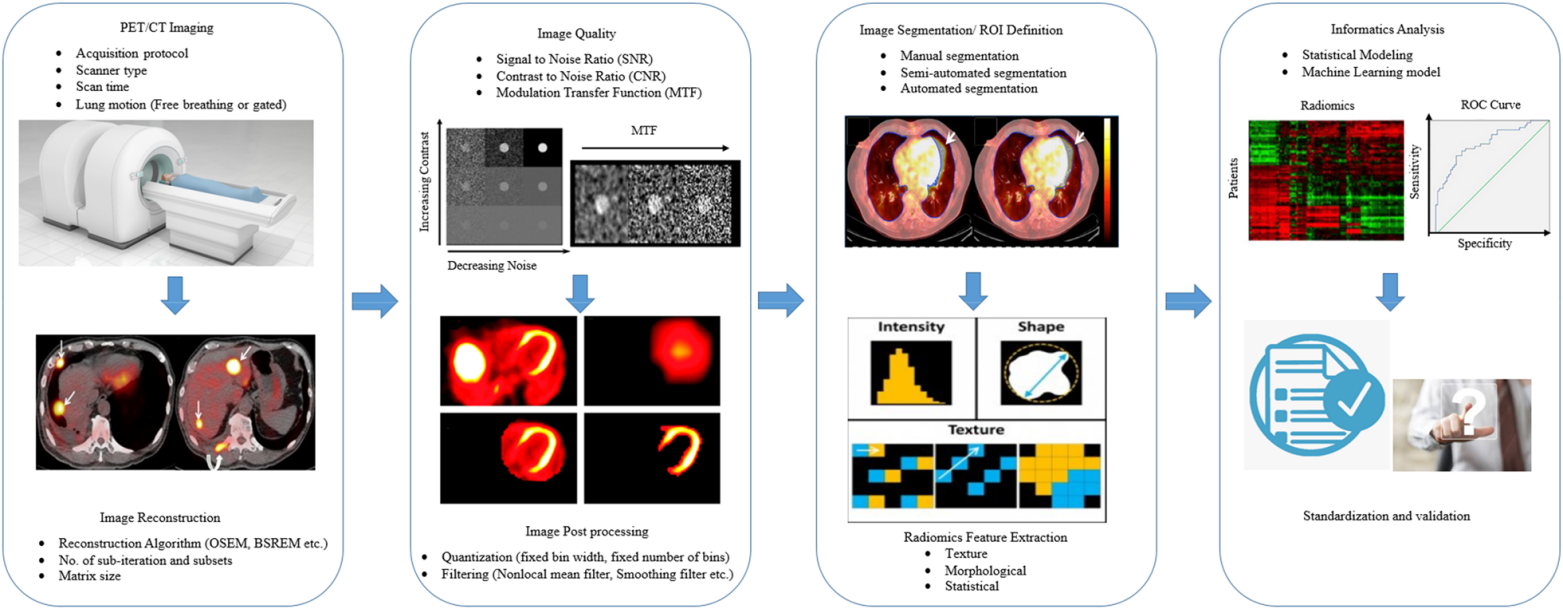
Workflow of radiomics texture analysis

Figure 2 shows the workflow of radiomics texture analysis. The workflow of texture analysis begins with image acquisition. Afterwards, the acquired image is reconstructed using different software platforms. During image reconstruction, filtering process includes sharpening and smoothing. Next, delineation of region of interest (ROI) also known as segmentation is performed where the location of tumour is defined. Textural features are extracted from the ROI and finally statistical model, or machine learning algorithm is developed. The ^18^F-FDG PET/CT-based texture traits correlated with regional reappearance and cause-specific survival of patients undergoing radiotherapy and forecasted disease-free survival in NSCLC patients after invasive surgery [6, 93, 94].

### Lung infection and inflammation detection and diagnosis

There are a number of studies emphasised on the oncological role of ^18^F-FDG PET/CT [44]. However, function of ^18^F-FDG PET/CT in analysing other medical conditions is also worthy of attention [26, 27, 95]. Differentiation of tuberculosis from lung cancer could be perplexing as both diseases share some similar phenotypic traits and consists of solid nodule [96, 97]. Critically malignant tumour such as lung cancer results in severe clinical condition than solid active pulmonary tuberculosis without early diagnosis [98]. In situation even worse, diagnostic error due to unsuccessful detection of tuberculosis from lung cancer could result in inconvenient, expensive let alone unnecessary invasive medical procedure. Contrarily, diagnosis delay of lung cancer would oversight required treatment leading towards uncontrollable tumour progression and fatal consequences [99]. SUV_max_, a metabolic PET parameter, might barely differentiate these two conditions as to vast aggregation of activated macrophages and lymphocytes promotes high ^18^F-FDG avidity in both conditions [100, 101]. Addressing these issue, a research team demonstrated that personalised and distinctive diagnosis of solid active pulmonary TB and solid LC might be performed using the radiomics nomogram [102].

Histoplasmosis a fungal infection frequently appears as pleural lesion on radiographic imaging as it resembles various lung cancer characteristics such as ring-shaped calcification layers on CT and higher avidity on ^18^F-FDG PET [103–105]. According to literature review, there are some limitation on identification of histoplasmosis nodule [105]. Identification of histoplasmosis in apprehensive lung effusion might be possible using the documented radiological features [103, 106, 107]. Previous studies suggested that radiomics might perform a complex work of identifying histoplasmosis selectively from lung cancer [103]. The work was based on the hypothesis that the nodule and surrounding perinodular parenchyma-based radiomic features might be able to differentiate suspicious histoplasmosis lung nodules and NSCLC precisely [103]. CT and MRI imaging were found to be incompetent for distinguishing post-surgical tumours from inflammatory sites [108–110]. In addition, glucose metabolism escalation due to soft tissue inflammation increases difficulty in distinction between these health conditions [111]. A limited number of investigation have addressed the potential of feature designation and framework design combination on radiomics analysis-assisted medical assessment [54, 72, 112]. Furthermore, harmonised machine learning would require systematic evaluation as contrasting features highly influence learning algorithms application.

Recently, COVID-19 has drove researchers towards the assessment of the potential capacity of radiomics in differentiating corona virus infection (COVID) from non-COVID [113, 114]. Radiomics feature extracted from CT image of COVID-19 patients was found to bear noticeable similarities with pneumonia-identifying radiomic features [113]. Focusing on this issue, a preliminary investigation showed there is a distinction between COVID-19 pneumonia and other pneumonias such as flu, bacterial and mycoplasma-dependent pneumonias that might be achieved by radiomic feature-based models [113]. In another study, COVID-19 and non-COVID-19 patients were classified using radiomics feature extracted from CT scan images of lung. They employed a dual machine learning technique to distinguish COVID-19 from non-COVID-19 patients [115].

## Evaluation of variables that affect radiomics features

**Fig. 3:**
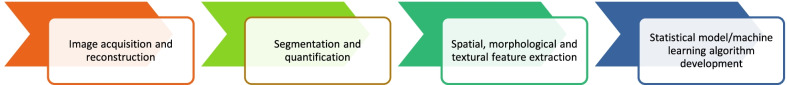
List of factors that affect radiomics feature analysis of lung diseases

Radiomics is the computable expression of the clinical imaging including PET, CT, PET/CT. Comprehensively, establishing quantitative feature-based classification and/or regression model is the purpose of radiomics. Sophisticated and subtle traces of diseases remain unnoticed when examining images in the conventional manner. Selection of reproducible and robust features is an arduous and prudent work, and radiomics has the potential to mine and detect those feature so that imaging modalities can be utilised thoroughly [37, 44]. In general, there are several factors that affect radiomics feature analysis of lung diseases as shown in Fig. 3. ^18^F-FDG PET/CT imaging protocol, post-processing techniques, image reconstruction, image quality, segmentation technique and the information analysis affect the radiomic feature analysis. The primary factor that affects radiomics is the ^18^F-FDG PET/CT imaging, i.e. the image acquisition protocol, scanner type, scan time, presence of lung motion [13].

Variation of protocol, scanner type and difference in scan time generate different outcomes even for the same subject. Lung motion influences image data collected from PET scan resulting artifact in texture features analysis as PET acquisitions take place typically for a few minutes in every bed position [61, 116–118]. The presence of lung motion induces noise (blur image) as a result the radiomics analysis becomes challenging. Respiratory gating is introduced to minimise the effect of lung motion. Accuracy of imaging increases by respiratory gating as blurring caused by breathing motion becomes negligible, but the application of respiratory gating in medical settings is not well established yet [119–121]. The 4D PET imaging might remove the effect of respiratory motion causing textural feature distortion present on 3D PET image [122].

However, the assumption of advanced textures might be interpreted from 4D-PET that requires future assessment and investigation [122]. Factors affecting CT acquisition such as metal artefacts [123], X-ray tube peak voltage and current [124], matrix size, and attenuation correction factor also impact PET radiomic feature quantification. Regardless of the apparent influence of these factors, robust analytical indications of the features could be perceived [122, 123, 125–127]. Reconstruction is one of the key factors that impacts radiomics analysis of the ^18^F-FDG PET/CT image [128]. There are two iterative reconstruction algorithms commonly used in clinical settings, and these are Ordered Subset Expectation Maximization (OSEM) and Block Sequential Regularised Expectation Maximization (BSREM). The BSREM has been proven to be more sensitive towards reconstruction [71, 192]. A study recently examined the role of deep learning for lung nodule detection in PET/CT as in almost 80% of cases higher ^18^F-FDG uptake by pulmonary nodule turns to become malignant. They suggested that deep learning may pose potential in this field and artificial intelligence performed better on BSREM than OSEM in detecting lesion, thus resulting in greater sensitivity [71]. Studies conducted by Yan et al. demonstrated that PET image reconstruction configuration variation does not alter some features such as normalised grey-level co-occurrence matrix, run-length matrix and size zone matrix [129, 130]. However, further elaboration is needed to determine the cause behind sensitivity variation of radiomic features [129, 130]. PET images have larger voxels than MRI and CT.

Thus, quantification of heterogeneity becomes biased [119, 131, 132] and it results in coarser tumour sampling. A large number of radiological features are sensitive to voxel dimension and so steady and equal voxel spacing is important for reproducing distinct image feature [45]. A study with the view of omitting the bias of voxel size in PET imaging concluded that the lower limit of volume for unbiased tumour sampling is 45 cm^3^ with the compromise of sensitivity of second-order entropy [132]. Contrast, resolution and noise are integral parts of any digital imaging technique. Image matrices such as signal-to-noise ratio (SNR) and contrast-to-noise ratio (CNR) are used to understand image quality. Radiomics analysis is affected by the low SNR and CNR of PET scan. In the case of PET imaging, CNR could be optimised by selecting the best possible segmentation method. In the case of SNR, PET images inherent high noise; therefore, achieving high SNR is always challenging. However, optimisation of scanner sensitivity, administered dose and scan time may lead towards high SNR. Application of time of flight (TOF) and point spread function (PSF) modelling play a vital role in optimising SNR and contrast. However, a limited number of studies have incorporated these two techniques as shown in Table 1.

**Table 1:** A summary of previous findings on potential feature exploration based on ^18^F-FDG PET/CT image of lung diseases

No.	Dose (MBq)	Scanner type	Scan time (min)	Respiratory gating	Reconstruction algorithm	Matrix size	PSF Modelling	Time of flight (TOF)	Image processing filter	Type of segmentation	Type of quantisation	Tumour size (cm^3^)	REF
1	3.70–4.81	Discovery ST4, GE Healthcare	16–12	No	OSEM	CT: 512 × 512	No	No	Exponential filter, square filter, square root filter, logarithm filter and wavelet decomposition	Manual	–	–	[42]
2	VD: 370 FD: 400	Validation DS: VCT-XT-Discovery, GE-Healthcare Feasibility DS: GE Discovery ST PETCT system (GE Healthcare, Waukesha, WI, USA)	–	No	OSEM	–	No	No	None	Automated	–	–	[50]
3	210–620	Discovery ST, GE Healthcare, Waukesha, WI)	–	No	OSEM	–	No	No	–	Manual	Equal-probability and Lloyd–Max quantisation	–	[55]
4	220 – 690	Philips GE PET/CT scanner (Philips Medical Systems, USA)	–	No	RAMLA	–	No	No	5-mm full-width at half-maximum Gaussian	Semi-automated	64 grey-level quantisation	–	[57]
5	350–550	Siemens Biograph 6 LSO (Siemens, Erlangen Germany) or a General Electric Discovery 690 (General Electric Healthcare, Waukesha, WI, USA)	–	No	Iterative, TOF, sharp IR	PET: 128 × 128, CT: 512 × 512	No	Yes	None	Semi-automated	PET: 64 bins from 0 to 25,CT: 400 bins from − 1000 to 3000	1.64 ± 0.78	[77]
6	340–450		24–28	No	OSEM	CT: 512 × 512 128 × 128	No	No	None	Manual	256 bins quantisation	< 3	[80]
7	370	ECAT EXACT 47 scanner (CTI/ Siemens, Munich, Germany)	–	No	OSEM	–	No	No	–	–	–	–	[86]
8	350–550	Siemens Biograph 6 LSO (Siemens, Erlangen, Germany), General Electric Discovery 690 (General Electric Healthcare, Waukesha, WI, USA)	–	No	Iterative, TOF, sharp IR	PET: 128 × 128, 256 × 256 CT: 512 × 512	No	Yes	–	Semi-automatic	Tonal discretisation (64 bins)	–	[94]
9	–	GE I PET/CT scanner (Philips),	–	No	RAMLA	–	No	No	5-mm full-width-at-half-maximum Gaussian	Automatic	–	–	[117]
10	370–555	General Electric Medical Systems, Waukesha, WI	35	Yes	OSEM	128 × 128	No	No	–	Automatic	–	–	[119]
11	618–814	Siemens Biograph PET/CT scanner (Siemens AG, Erlangen, Germany)	Non-gated: 18–35 Gated: 20–30	Yes	3D PET: OSEM, 4D PET: OSEM	No-gated: 168 × 168 Gated: 256 × 256	No	No	Non-gated: 7 mm full-width half- maximum Gaussian Gated: 5 mm full-width half-maximum Gaussian	–	32 discrete values quantisation	–	[122]
12	223–690	GE I PET/CT scanner (Philips)	–	No	RAMLA	–	No	No	5 mm in full width at half maximum Gaussian	Automatic	–	–	[138]
13	550	Philips GE I PET/CT, Philips Health Care, Cleveland, Ohio, USA	–	No	RAMLA	PET: 144 × 144, CT: 512 × 512	No	No	–	Manual	–	0.7–5.8	[6]
14	150–310	Discovery MI, GE Healthcare	15–20	No	OSEM BSREM	256 × 256	Yes	Yes	6.4-mm Gaussian filter with time-of-flight and PSF, and BSREM with a beta-value of 450	Manual	–	≤ 2	[71]
15	555	GE discovery LS 4 PET/CT scanner	–	No	OSEM	128 × 128	No	No	8 mm full-width at half-maximum Gaussian	Manual	256-bin discretisation	–	[76]
16	350 – 450	Biograph 16 Siemens Medical Solutions	20–25	No	OSEM	PET: 128 × 128, CT: 512 × 512	No	No	5 mm full-width at half-maximum Gaussian	Manual	–	1.7–6.8	[81]
17	–	Biograph 16 PET/CT scanner	18–21	No	OSEM	164 × 164	No	No	–	Manual	64 bins quantisation	1.7 – 6.8	[93]
18	270–410	Biograph mCT scanner (Siemens, Germany)	–	No	OSEM	–	Yes	Yes	4-mm full-width-at-half-maximum Gaussian	Manual	–	> 3	[102]
19	440 ± 2.0	GE Discovery STE PET/CT Scanner, GE Discovery 600 PET/CT Scanner	–	Yes	OSEM	–	No	No	4.29 mm, 7 mm, or 10 mm full-width-at-half-maximum Gaussian filter	Automated	–	–	[123]
20	370	GE Discovery VCT scanner (Waukesha, WI)	–	No	2D PET: OSEM 3D PET: Iterative- Vue Point algorithm	2D: 128 × 128 3D: 256 × 256	No	No	3 mm, 5 mm, 6 mm post-filtration width	Semi-automated	–	–	[126]
21	229.4 ± 22.2	Biograph 64 mCT scanner (Siemens)	–	No	FBP and OSEM	256 × 256,128 × 128	Yes	Yes	2.5 mm, 3.5 mm, 4.5 mm, 5.5 mm full-width at half-maximum Gaussian	Semi-automated	–	< 5	[127]
22	740	Siemens Biograph PET/CT scanner	21–40	Yes	OSEM	3D: 168 × 168 4D: 256 × 256	No	No	3D scan: 7 mm full-width at half-maximum Gaussian 4D scan: 5 mm full-width at half-maximum Gaussian	Automated	–	< 3	[147]

Higher SNR can be attained by TOF resulting in heterogeneity generated by noise and improving image quality. On the other hand, higher resolution is obtained by PSF modelling as it models the matrix physical processes, producing detailed structures within lesion. The post-processing techniques involve smoothing by averaging the pixels, application of Gaussian filters to improve the scan image quality, image noise regulation and image enhancement by the virtue of histogram equalisation, deblurring and resampling [55, 133]. Quantisation performed for image-acquired noise-suppression is an important step of tomographic modification for traceable calculation of texture features, which also impacts radiomics feature analysis [45].

Conventionally, fixed number of bins and fixed bin width are the two approaches of quantisation, and both techniques come with their specific characteristics accommodating their use per requirement [45, 134]. The effect of segmentation is well recognised as the selection of segmentation method determines the balance between accuracy and reproducibility. In the case of manual segmentation, inter- and intra-observer variability is always present without question [135–139]. Feature nomenclature and feature extraction guideline are yet to be established. Therefore, variation is present in extracted features causing variation of radiomics analysis. Three types of features are mainly extracted from the ROI. These are texture, morphological features and statistical features. Texture provides information in the spatial arrangement of intensities in an image. Texture feature computation involves dataset comparison and rotationally even voxel spacing distribution [45].

Common set of textural features are those derived from the grey-level co-occurrence matrix (GLCM) and grey-level run-length matrix (GLRLM). Morphological features describe the shape of the delineated ROI and properties including its volume, maximum diameter, maximum surface, tumour compactness and sphericity. Statistical features include mean, median, skewness, kurtosis, uniformity and entropy. Another key factor that influences radiomics study is statistical calculation [13]. Correction for multiple testing is one of the important steps of accuracy of feature while working with large dataset [119, 140]. Surprisingly, a retrospective systemic review by Alic *et al*. showed that a significant number of radiomics trait become statistically inconsequential when the correction factor is applied [141].

Validation and standardisation of radiomics features pose significant challenge [142–144]. Some studies comprise validation-level limitations such as inadequate statistical study such as asynchronous *p*-value for multiple tests, insufficient independent validation dataset resulting in biased discovery rates [7, 145]. Validation of radiomics approaches requires ample multicentre datasets [7]. Overstatement of positive results against negative ones is also another crucial factor [146, 147]. Quantification of radiomic feature with identical names might have different implementation due to the lack of standard definition in radiomic studies. For example, calculation of GLCM could be done by averaging matrix values of 13 distinct directions or a single matrix encompassing tumour co-occurrence values in all 13 directions [131]. Indistinct feature terminology and feature definition variation caused by different operating systems (MaZda1, CGITA2, IBEX3, LIFEx4, MITK Phenotyping5, RaCaT6, CERR radiomic extension7 and Pyradiomics8) also affect the radiomics analysis [148]. These issues have been addressed by the IBSI initiative [148].

## Importance of ^18^F-FDG PET/CT image biomarker standardisation

Radiomics has received much attention and interest in the field of medical science. Nonetheless, reproducibility and validation of the published work are still a big challenge [44, 149–152]. The absence of unanimously recognised reference values and definitions has hampered clinical use of ^18^F-FDG PET/CT image biomarker. Furthermore, there is lack of uniformity of the image processing platforms required to analyse features [153–155]. Manipulation and assessment of a single image set in two different software platforms result in dissimilar feature values [156]. Variation of imaging procedure, ^18^F-FDG activity, image reconstruction, data comprehension and uptake time are significant [128, 157, 158]. Reproducibly has been challenged frequently as there is a lack of detailed report of the reproducibility of the experiments. There are various factors including the absence of open-source data and standardised protocol that limit the reproducibility studies of the radiomics features [159–161]. The situation can be solved by standardisation of the radiomics features definition with supportable references and coherent execution of image assessment strategies for feature quantification [103, 154, 156, 162]. The mainstream quantification of ^18^F-FDG PET/CT is accomplished using the quantitative index of tracer uptake called SUV.

Quantification by SUV is well recognised despite having variance of factor [64]. In the interest of strengthening, the application of ^18^F-FDG PET/CT as imaging biomarkers guidelines on tumour imaging using ^18^F-FDG PET/CT has been published and revised [163, 164]. Currently, it is well understood that harmonisation of imaging modalities is vital alongside standardising imaging performance for standardising computation of ^18^F-FDG PET/CT as biomarker [64]. Reproducibility and validation of radiomic features are hard to achieve without standardising the software platforms used across different research facilities. A study on the level of agreement between IBSI guideline and the Image Biomarker Explorer (IBEX) that is an open-source radiomic software was performed alongside development and validation of S-IBEX [165, 166]. The software platform achieved validation by employing the five different pre-processing configurations proposed by IBSI [143, 166].

**Fig. 4:**
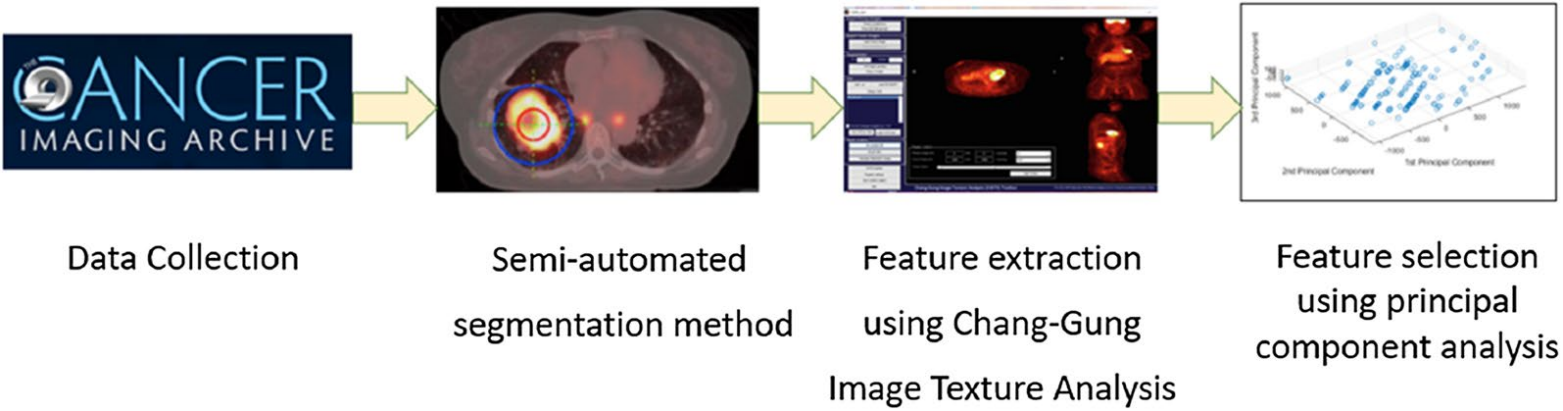
Adenocarcinoma in non-small cell lung cancer (NSCLC) detection using radiomics [167]

From the literature, it is well understood that application of radiomics in lung disease management has been able to attract great interests for the past few years. For example, a study was performed on feature selection to identify adenocarcinoma histologic subtype present in non-small cell lung cancer (NSCLC) [167]. Figure 4 illustrates the detail procedure of feature selection. In this study, the initial PET/CT data were collected from The Cancer Imaging Achieve [168]. Semi-automated segmentation was applied on the images to delineate the region of interest. Chang-Gung image texture analysis (CGITA), an open-source platform, was then used for extraction of textural features form the segmented ROI. Principal component analysis (PCA) was performed in MATLAB before feature selection, to minimise the feature space and maximise the relevant information. The feature selection criteria were set to select features having a coefficient with one principal component with the major variance and the normalised value retaining 99% of the variability.

**Fig. 5:**
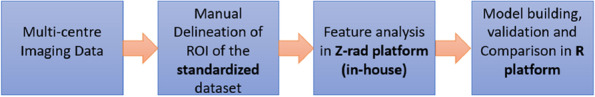
Workflow PET radiomics model for prediction of event-free survival in locally advanced NSCLC using multicentre datasets [169]

In another study, a prediction outcome of locally advanced NSCLC was investigated [169]. The detail workflow is illustrated in Fig. 5. This study highlighted the importance of PET acquisition standardisation. They emphasised the role of preselection in the case of determining robust radiomic features. The method started by extracting 1404 radiomic features. The dataset included either pre-treatment ^18^F-FDG PET scans of stage IIIA/N2 or IIIB NSCLC patients. In this prospective study, robustness was determined against tumour motion, delineation variation and attenuation correction. Finally, the training of regression models was performed using standardised imaging. Validation was done in two ways. It includes separate single-centre dataset and fivefold cross-validation. The performance of the model was denoted by area under the receiver operating characteristic curve (AUC).

**Fig. 6:**
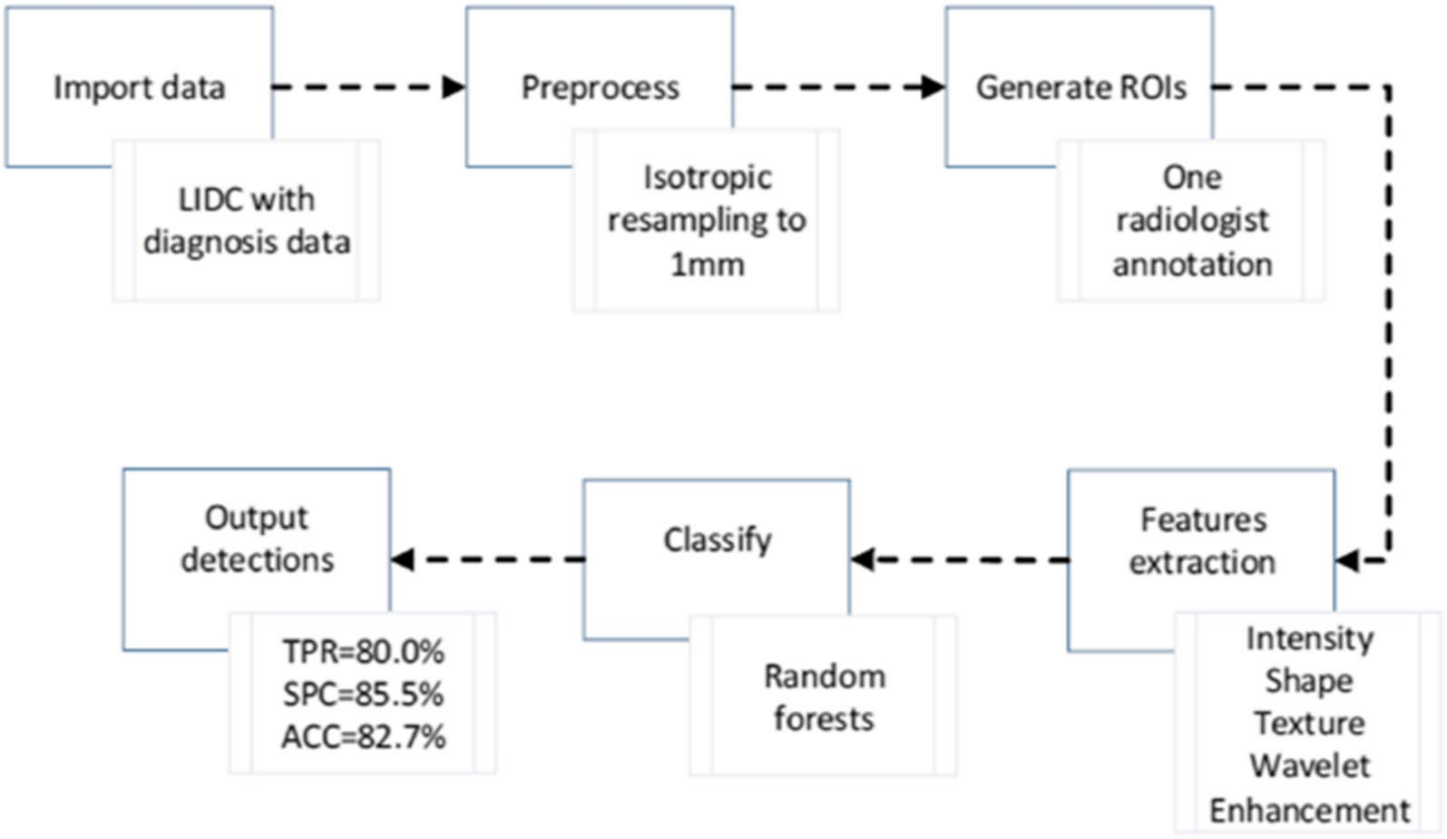
Workflow of automatic lung nodule classification with radiomics approach [170]

Another study proposed a computer-aided diagnostic (CAD) method for identifying the benign and malignant lung cancer utilising radiomics from CT images [170]. The method attained 82.7% accuracy in distinguishing between benign and malignant primary lung nodules. The intensity, heterogeneity information and shape of the suspected nodules were quantified using 583 features, at multi-frequencies. Random forest method was then applied to identify benign or malignant nodules by analysing all these features. The step-by-step flow chart is shown in Fig. 6.

**Fig. 7:**
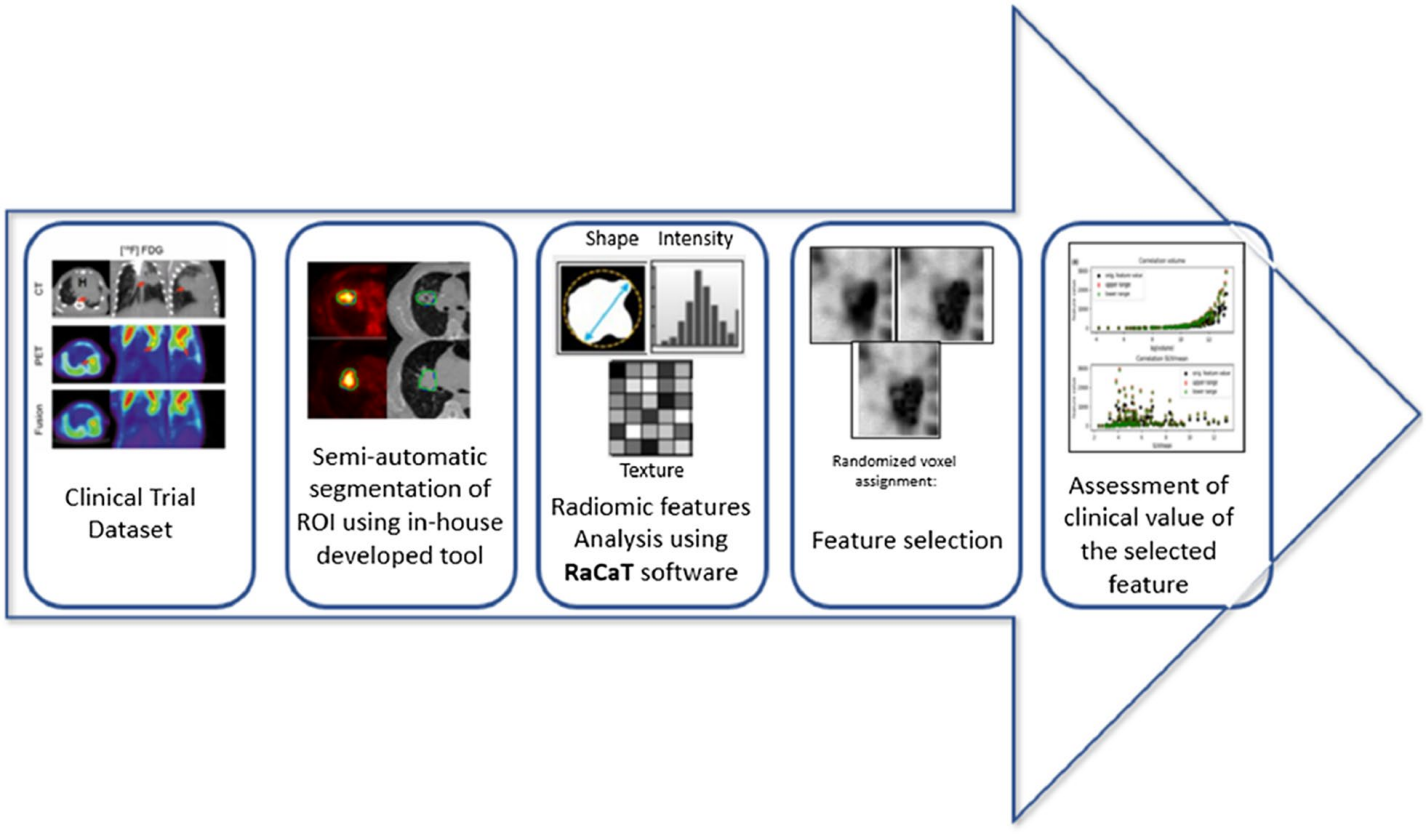
Workflow of feature selection procedure for reproducible textural feature identification describing relevant texture and independent of conventional PET metrics [191]

In addition, Fig. 7 illustrates another robust feature selection method for NSCLC diagnosis [171]. The method is free of false-positive findings and overfitting. In this method, a semi-automated segmentation method was applied before extracting radiomics features. The features were then analysed using an open-source platform, RaCaT which follows the IBSI [166]. Finally, data analysis was performed using Python.

## Challenges to overcome in ^18^F-FDG PET/CT image biomarker standardisation

**Fig. 8:**
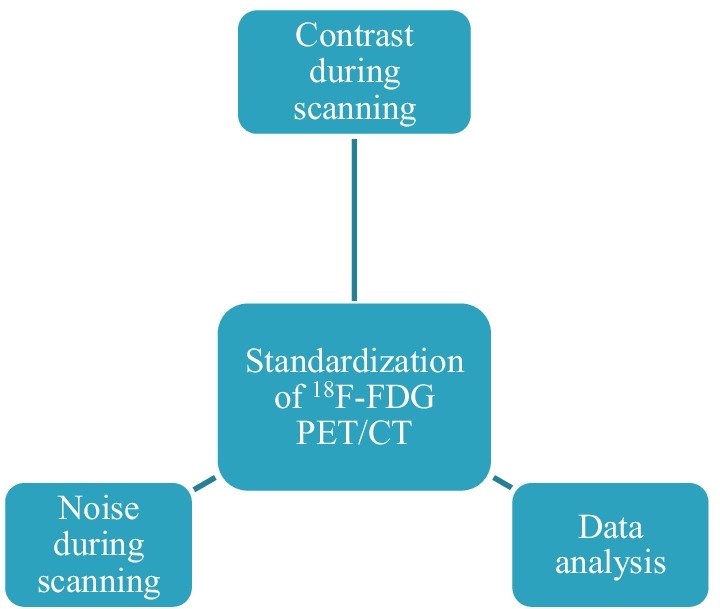
Factors impacting ^18^F-FDG PET/CT feature standardisation

Standardisation is the prime solution of any challenges faced in enhancing quality and safety of clinical care. In this era of technological advancement, when new findings meet reality and new information gets stacked with every passing second, standardisation of ^18^F-FDG PET/CT is far from being easy. Optimisation of feature calculation is a significant step towards reproducible radiomics. In radiomics analysis, image acquisition, reconstruction and segmentation present considerable influences for heterogeneity [16]. Characteristics that can be replicated using optimised radiomics tools from the same image can still lack reproducibility in multicentric or multi-scanner configuration unless the parameters associated with image acquisition, reconstruction and segmentation attain standardisation and harmonisation [159, 172]. Key factors impacting ^18^F-FDG PET/CT feature standardisation are illustrated in Fig. 8.

As the figure shows, standardisation of features can be achieved using well-defined combination of image characteristics (resolution, noise) and data analysis method. Change of any of these factors results in change of feature calculation. During the initial stage of lung disease, the change in the molecular level takes place before anatomical changes happen. This change at molecular level is responsible for the heterogeneity. When ^18^F-FDG PET/CT scan is performed for detecting the biological heterogeneity, mechanical heterogeneity is introduced by the scanner. Image resolution, noise and the data analysis technique induce the mechanical heterogeneity. By optimising resolution and noise during ^18^F-FDG PET/CT imaging and harmonising, the data analysis technique will eventually result in standardisation of ^18^F-FDG PET/CT.

Accuracy, feasibility and precision are the must meet criteria for establishing biomarker and moving towards standardisation. In 2001, the biomarkers definition working group defined the biomarker as objectively measured and evaluated characteristic as an indicator of homeostatic biologic, pathogenic processes, or drug responses duding treatment [173]. Thus, biomarker is the assertive diagnostic and treatment standard which characterises biological and functional activities [64]. It shows that image feature can be acknowledged as biomarker when it is standardised. Accuracy is defined by the correctness of a measurement and determined by comparing the measurement against the true or accepted value. From the view point of medical science, accuracy is the ratio of true positive and true negative in all calculated cases under a specific investigation [174].

Accuracy in the case of radiomics can be assured by comparison between computed values and mathematically or theoretically correct value, which requires consistent and focused effort. Feasibility denotes that the image feature contains logical, analytical and extrapolative value. Radiomics being a computational analysis, overwhelming number of features are obtained by image processing and so selecting feasible features from data large cohort of data is of highly challenging. Precision in achieved by meeting the criteria of acceptable fidelity, repeatability and reproducibility. The systemic review conducted by Traverso et al. confirmed that the repeatability and reproducibility of radiomic features depend on acquisition, reconstruction algorithm, preprocessing and software platform employed for computing the features [159]. Reproducibility is one of the big challenges in ^18^F-FDG PET/CT radiomics studies. Factors affecting the reproducibility of biomarkers have been unanimously agreed [154, 159, 163, 172, 175]. In multicentre clinical trials, incorporation of ^18^F-FDG PET biomarkers and treatment response would be impossible without calibration and optimisation of the quantitative ^18^F-FDG PET parameters. A study conducted with the aim of ^18^F-FDG PET/CT uptake test–retest reproducibility in cancer patients based on multicentre qualification processing found that ^18^F-FDG PET/CT scanner quality and settings may result in significantly reproducible test–retest tumour SUV measurements [176]. However, kinetic behaviour of the tracer uptake is not considered in SUVs [177].

Standardisation of radiomics analysis depends on the optimisation of the PET image quality parameters such as contrast-to-noise ratio (CNR) and signal-to-noise ratio (SNR) [178]. However, the PET scan inherently has low SNR and CNR compared to other diagnostic imaging modalities [179]. Reconstruction algorithm types and parameters also impact the radiomics features. Likelihood expectation maximization (MLEM) or ordered subset expectation maximization (OSEM)-type algorithm are highly affected by the minute changes of initial data. Consequently, the resulted outputs become noisy as the iteration converges. To minimise this effect, the iteration is typically stopped before it reaches full convergence, which may introduce bias in the reconstructed images [180]. Block sequential regularised expectation maximization (BSREM), which includes an edge-preserving penalty term, can be used to obviate all these problems. BSREM algorithm achieves optimal SNR by using the penalty term as it employs low smoothing in higher activity areas (such as tumour) as well as in neighbourhood with the high-intensity edges and high smoothing in lower activity regions (such as background) [180].

In addition, a study was performed by Gabriel Reynés-Llompart et al., to prove the promising role of a radiomics approach to assess image quality of abdominal PET imaging by using new reconstruction algorithms with BSRM methods and testing the utility of a radiomics approach. This study found for the OSEM + PSF and especially for the BSRM reconstructions; the image quality parameters presented only at best moderated correlations with the subjective image quality. None of the studied parameters presented a good predictive power for image quality, while a simple radiomics model increased the performance of the image quality prediction [192].

Statistical method application could narrow down the influence of cohort size on radiomics features [181], and artificial intelligence would improve conversion between reconstruction kernels in CT imaging [182]. Incorporating AI into the image analysis field comes with its challenges. Computer-aided detection system always generates false-positive results, which increases the workload of false finding elimination for radiologists and physician. The task is also time-consuming though it does not affect the patient care to a great extent. Medical imaging has still to collect ample amount of data so that AI algorithm can be trained rigorously. This challenge can be dealt by introducing neural network-based transfer learning. Successful implementation of neural network relies on unbiased and large training set and assimilation of different settings [183, 184].

## Overview of standardisation methods of PET image biomarkers

The advancement of ^18^F-FDG PET/CT standardisation in oncologic imaging is noteworthy [10, 148, 149, 185, 186]. Research works dedicated towards developing novel imaging biomarkers have been proposed [4, 187, 188]. Standardisation methods have been employed during the scan time (image reconstruction modification, scan framework reformation), patient level (blood glucose level regulation and amelioration, supervising tolerable delay time of radiotracer dose and uptake) and image post-processing level (z-score, transformation method) [10, 186]. Alleviation from the undesired nonetheless unavoidable image acquisition interrupters, namely body weight, radiotracer uptake interval, partial volume [4], is the goal of the studies focusing diagnostic and prognostic image standardisation.

In recent times, specific guidance for addressing the limitations faced in application of radiomics analysis has also been published [154]. Study on steadiness of nearly 100 radiomic features and inter-observer variability in lung tumour identification [123] showed that the PET-based stable features were also robust to interobserver variability. From their observation, they suggested that poorly reproduced features might also be sensitive to other factors as well [122]. Scientists have also confirmed the invariability of some features, regardless of the reconstruction configuration applied [61, 121]. Standardisation is required for image acquisition, reconstruction, segmentation and feature calculation. In this present work, we focused on standardisation initiative by the international collaboration such as Image Biomarker Standardisation Initiative (IBSI) alongside post-acquisition standardisation method and histology standardisation techniques.

**Fig. 9:**
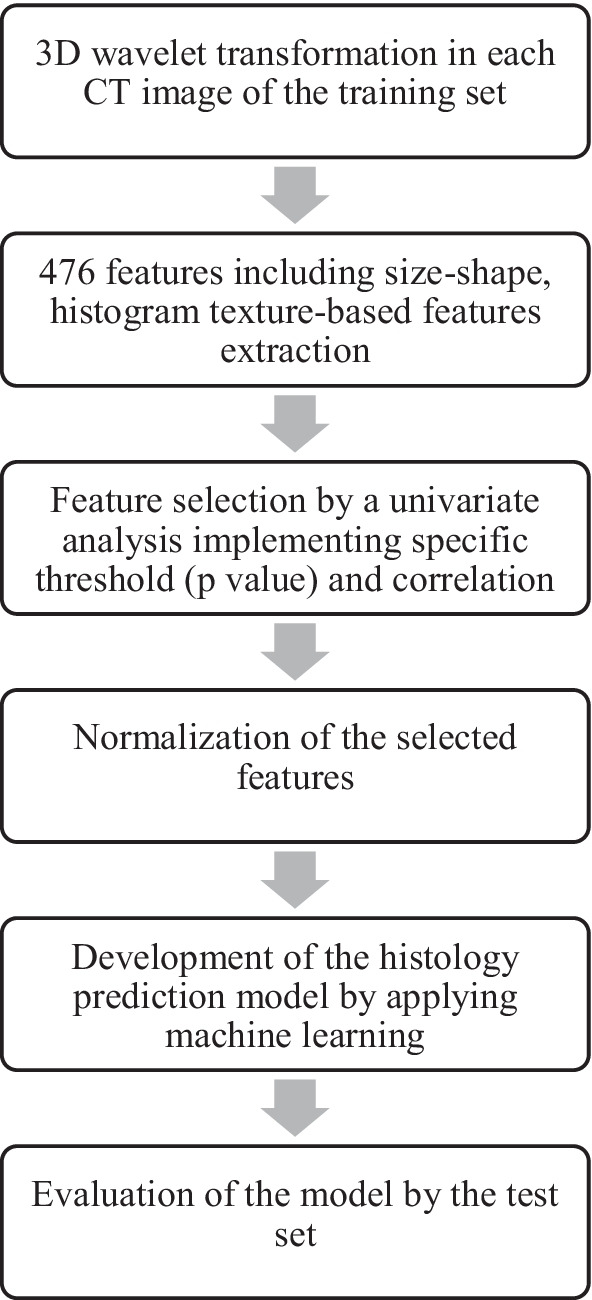
Flowchart of feature extraction study based on CT images performed by Haga, Akihiro et al. [173]

Figure 9 shows a flowchart of standardisation process applied on the collaborative investigation by the researchers of Tokushima university and the university of Tokyo hospital jointly [186]. They were able to successfully upgrade the accuracy of histology extrapolation. The study was performed on CT image sets (training set and test set) of patient having confirmed adenocarcinoma, squamous cell carcinoma and NSCLC [186]. They considered the inter-observer variation by considering four segmentations of a tumour. In the first step, CT images were transformed into three-dimensional wavelets. Then, 476 features were generated from the raw and the transformed images. Next, using univariate assessment of a fixed threshold, feature selection was performed. To eliminate the effect of imaging condition the team performed normalisation of features. Random forest model was used to build the histology prediction model, and its performance was verified by test cohort specificity, sensitivity, accuracy and receiver observed characteristic curve. However, the standardisation technique built, should be performed on large cohort of patients for further validation.

**Fig. 10:**
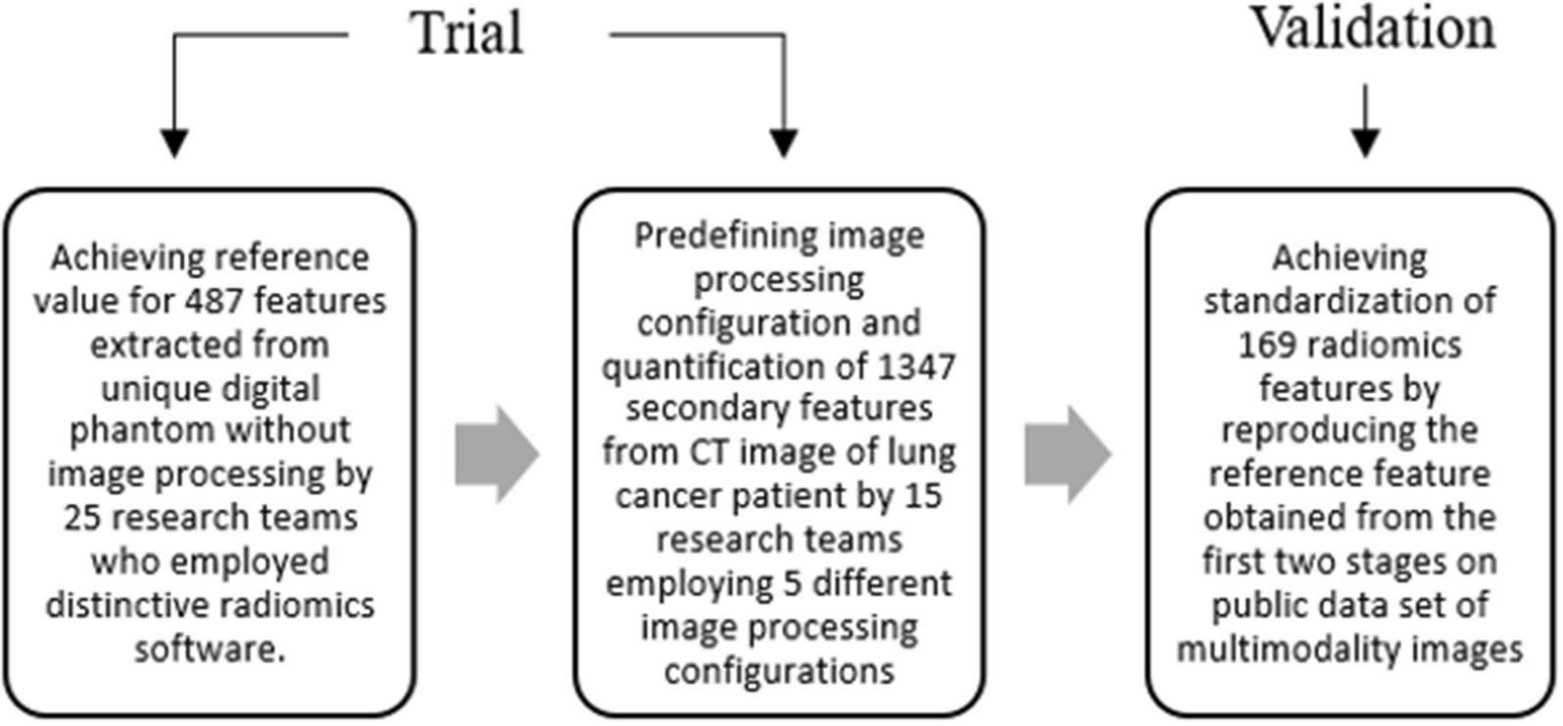
Flowchart of validation study by Zwanenburg, Alex et al., overview [145]

In 2007, Radiological Society of Northern America organised the Quantitative Imaging Biomarkers Alliance for systematic validation and standardisation of a number of radiological biomarkers [4]. The achievement of standardising 169 radiomics feature and standardising image processing framework by this researcher alliance is definitely a breakthrough towards automation of disease diagnosis. They accomplished the enormous work of defining 174 radiomic features and efficaciously reproducing thus standardising 169 features. Figure 10 demonstrates the workflow of the study.

The study was performed in three stages. Digital phantom with specific ROI mask (74 voxel) was used as the dataset during the first phase. In the next phase, the dataset was taken from open-source CT images where the ROI was defined by the segmented gross tumour volume. Research groups calculated feature values from the ROI directly and applied predefined image processing pathway as per requirement. Standardisation of the computed values was achieved by comparing the degree of concurrence and stability of the concurrence. In the first two stages, the feature reference values were achieved through iterative filtering level of concurrence. The features calculated in these two phases was recognised as standard only when the degree of concurrence was high enough. In the third stage, dataset of patients diagnosed with soft-tissue sarcoma was selected from Cancer imaging Archive consisting of multimodality imaging (T1-weighted MRI and ^18^F-FDG PET/CT). Similar to the previous phase, the images were accompanied by segmentation of the gross tumour volume. In this stage, the standardised features (achieved from the first two phases) were validated by reproducing the features using predefined image processing configuration on the dataset. Finally, IBSI achieved the standardisation of 169 features out of 174 features examined in this research study. However, the study excluded uncommon features and traits such as fractals and image filters for feasibility purpose.

**Fig. 11:**
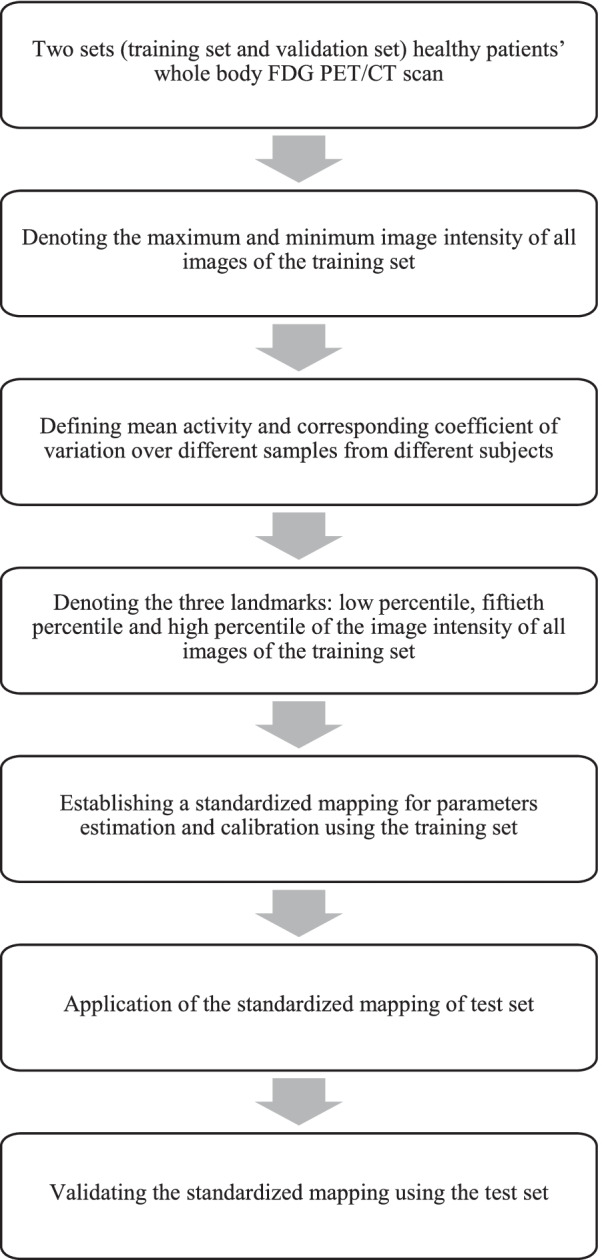
Flowchart of establishment of standardised mapping for whole-body FDG PET/CT scan study by Mortazi et al. [10]

A research team recently presented a post-acquisition standardisation workflow. Their proposed principle is based on the modified MRI standardisation method recommended by Nyul et al. [189, 190]. The workflow followed during this study is illustrated in Fig. 11. Two sets of ^18^F-FDG PET/CT scan data were utilised in this study as the training set and validation set. First, a standardised intensity scale was defined for the image set. This was achieved by initially computing the low percentile, 50^th^ percentile and high percentile intensities and mean values of these intensities from the training image dataset. Then, the intensity of images from test dataset was mapped nonlinearly in the mean value interval obtained from the training dataset.

Performance of the standardisation process was determined by implementing the coefficient of variation of mean metabolic activity and coefficient of variation of mean activity computed from the training dataset. One of the utilities of this technique is that it is free from the effect of patient and image acquisition parameters. The study result showed significant decrease of the mean metabolic activity coefficient variation was achieved by standardised PET (sPET). Additionally, sPET was proven to be superior to conventional standardisation methods such as SUV and z-score normalisation [10]. However, the validation data set adopted in the study was healthy cohort of patient. The developed sPET scheme should also be applied on diseased patients in clinical trial to further investigate its feasibility.

## Conclusion

The micro-level changes in lung disease such as tumour, infection or inflammation progresses cannot be detected by CT scan alone. ^18^F-FDG PET/CT diagnosis carefully examines detailed and diverse cell anomalies in the field of biology. Nevertheless, low-grade resolution and irregular noise of PET images upholds added methodological boundary. In the field of tumour treatment response assessment, the use of ^18^F-FDG PET/CT is not as common as CT. This is because the globally approved PET equivalent of CT-Based Response Evaluation Criteria in Solid Tumours guideline is yet to be established. Application of radiomics might enhance the diagnostic capability of the imaging techniques as it extracts a large number of quantitative features from the images which otherwise remains unattended. Biologic changes at the molecular lever can be traced back by intelligent assessment of the computed features. Employment of ^18^F-FDG PET/CT radiomics, especially texture analysis in lung abnormalities management, directs its gradual steps towards a patient-specific approach of lung diseases management.

Recently, application of ^18^F-FDG PET/CT radiomics is also being applied for lung infection and inflammation diagnosis. However, numerous numbers of features and various techniques of feature extraction have raised tremendous complexity. This complexity can only be removed by standardisation of radiomics analysis. Hence, introduction of radiomics in the medical practice is impossible without standardisation and harmonisation through sufficient and convincing scientific evidence. Importance of standardisation, reproducibility, and validation of radiomics in clinical trials cannot be overlooked.

To achieve the ultimate goal to employ radiomics analysis as an integral part of the routine medical diagnosis and prognosis, validation of its robustness across reconstruction algorithm and parameters is crucial. However, the absence of appropriate cross-validation of the radiomics studies till date raises the concern of false-positive results. Radiomics textural features are a set of numeric and their interpretation by human, which are often difficult. Elucidation of these feature statistics is not beyond mistakes, e.g. assumption of correlation implies the causation, misinterpretation of correlation, over generalisation [161]. Acceptance of appropriate radiomic features will only be achieved once these challenges are properly addressed. For the time being, comparison of the findings across different studies is unattainable due to different protocols and practices. Only the establishment of a uniform prognostic and predictive factors of feature analysis can promote the transition of radiomics into the field of clinical practice. Basically, the available studies on radiomics are mainly retrospective and hence demonstrate the perception of radiomics. Adaptation of prospective research is essential to establish radiomics into the medical support system. Acceptance of radiomics can only be achieved upon proper addressing of these challenges.

The standardisation and quantification of ^18^F-FDG PET/CT radiomics will increase its potential field of application even more. The combination of artificial intelligence and machine-learning techniques with radiomics research will hold the ground of disease diagnosis and treatment evaluation robustly and speed up medical translation.

## Data Availability

All data and materials concerning this work are available in the submitted manuscript.

## Abbreviations

^18^F-FDG PET/CT: 18-Fluorine-fluorodeoxyglucose positron emission tomography/computed tomography
CT: Computed tomography
GLCM: Grey-level co-occurrence matrix
GLRLM: Grey-level run-length matrix
IBEX: Image Biomarker Explorer
MRI: Magnetic resonance imaging
NSCLC: Non-small cell lung cancer
PET: Positron emission tomography
PET/CT: Positron emission tomography/computed tomography
ROI: Region of interest
sPET: Standardised PET
SUVmax: Maximum standard uptake value
