# Correction: A review on advances in 18F-FDG PET/CT radiomics standardisation and application in lung disease management

**DOI:** 10.1186/s13244-022-01186-8

**Published:** 2022-02-28

**Authors:** Noushin Anan, Rafidah Zainon, Mahbubunnabi Tamal

**Affiliations:** 1 Department of Biomedical Imaging, Advanced Medical and Dental Institute, Universiti Sains Malaysia, SAINS@BERTAM, 13200 Kepala Batas, Pulau Pinang Malaysia; 2 School of Physics, Universiti Sains Malaysia, 11800 Pulau Pinang, Gelugor Malaysia; 3 Department of Biomedical Engineering, College of Engineering, Imam Abdulrahman Bin Faisal University, PO Box 1982, Dammam, 31441 Saudi Arabia

## Correction to: Insights into Imaging (2022) 13:1-22 10.1186/s13244-021-01153-9

The original article mistakenly omitted a Funding source which has since been re-instated.

